# Viral Neoliberalism: The Road to Herd Immunity Still A Rocky One

**DOI:** 10.1177/00207314221131214

**Published:** 2022-10-09

**Authors:** Jens Holst

**Affiliations:** 38883 Fulda University of Applied Sciences – Health Sciences, Fulda, Germany

**Keywords:** pandemic, syndemic, COVID-19, viral neoliberalism, marketisation, inequality, philanthrocapitalism

## Abstract

The objective of this article is to assess the dominant global economic system and the resulting power relations from the perspective of the strategies used worldwide against the SARS-CoV-2 pandemic. The predominantly biomedical approach has not sufficiently taken into account the actual dimension of COVID-19 as a syndemic. While the much longer-term pandemic caused by the neoliberalism virus has not been systematically considered by public and global health scholars in the context of COVID-19, it exhibits essential characteristics of an infectious pathogen, and the symptoms can be described and detected according to biomedical criteria. Even more, the severity of leading symptoms of neoliberalism such as growing inequities calls for immunization campaigns and ultimately herd immunity from viral neoliberalism. However, achieving worldwide immunity would require an anti-neoliberal vaccine, which is extremely challenging to develop vis-à-vis the power relations in global health.

## The Neoliberalism Syndemic

For over two years, coronavirus disease 2019 (COVID-19) has been the dominant topic worldwide. Everything revolves around how, when, and by what means this pandemic can be ended. Several authors consider COVID-19, meanwhile, a syndemic rather than a pandemic^1^ due to the interplay between the political, economic, and social distribution of power, the unequal distribution of health risks and resources, and the interactions of overlapping diseases.^2,3^ Scholars have applied the concept of a syndemic in relation to the Human Immunodeficiency Virus/Acquired Immunodeficiency Syndrome (HIV/AIDS)^4^ and other widespread infections such as tuberculosis,^5^ as well as to chronic diseases such as diabetes, obesity, and the metabolic syndrome.^6^ The complexity and multilayered nature of syndemics has not yet been adequately taken into account in managing the COVID-19 pandemic and even less so in the concept of health security; likewise, the relevance of the ubiquitous disease symptoms caused by the neoliberalism virus as one overwhelmingly important contributor to syndemics has not been sufficiently addressed so far.

Some scholars previously coined the term “neoliberal epidemics,” arguing that health can be best understood as the direct consequence of the unequal distribution of power and resources. Neoliberal economic and social policies in many places have widened health inequalities and often imperiled past gains in people's health status, and these effects depend on a set of interdependent determinants, which spread so quickly across space and time that they would be considered as an epidemic if traceable pathogens were involved.^7^ By analogy, the classification of COVID-19 as a syndemic has emerged among global health scholars,^1,8–10^ with a main focus on the overlap with other diseases that cause a high burden on populations. To the author's knowledge, the COVID-19 syndemic has not yet been explicitly linked to the concept of viral neoliberalism, which has been defined and analyzed for quite some time. This article aims to enrich the hitherto marginal scientific debate about the neoliberal syndemic among public health and global health scholars by demonstrating the relationship between COVID-19 and the neoliberalism virus from biomedical and clinical perspectives. On one hand, the link between the two viral infections is reflected in the obvious commonalities between the two pandemics; on the other hand, it represents an attempt to make the inconsistency of some COVID-19 measures comprehensible to non-medical professionals.

The aim of this article, written from a (global) health perspective, is to sensitize experts from different fields of social and human sciences for the medical approach to the COVID-19 pandemic and the lessons to be drawn for pushing back other viral pandemics, such as neoliberalism, which would in turn help drive back outbreaks of other viruses. The article is based on the analysis that the neoliberalism virus is likely to be more dangerous and more virulent in the longer term than the Severe Acute Respiratory Syndrome Coronavirus-2 (SARS CoV-2) that has been propelling the world for more than two years now.

The neoliberalism virus has been spreading and infecting governments, as well as academia, media, and think tanks, worldwide for a much longer period of time—that is, for decades.^11^ Likewise, the clinical manifestation of the virus in the form of neoliberal practice has long been present in international agencies such as the International Monetary Fund, World Bank, and World Trade Organization, as well as the health-relevant specialized agencies of the United Nations (UN), including the United Nations International Children's Fund (UNICEF) and the World Health Organization.^11^ Viral neoliberalism is in fact a suitable metaphor for two reasons: On one hand, neoliberalism shows the typical patterns of a contagion-based spread causing certain symptoms, which can be described as the neoliberalism syndrome; and secondly, it proves to be highly adaptable to changing conditions, to which it responds with ever-new mutants.^12,13^

From a clinical perspective, the neoliberalism virus invades the human cerebrum in the disguise of common beliefs, myths, half-truths, and fake news, which are based on widely unproven theories of free markets and free trade^11,14^ but tend to reflect individual and subjective perceptions and correspond with everyday experiences. Unlike SARS-CoV-2, the neoliberalism virus does not spread exclusively through direct contact with infected persons. Transmission mostly occurs through exposure to avid economists, ubiquitous think tanks, media, and other infected sources.

## Pathophysiology of the Neoliberalism Virus

Neoliberalism is the commonly used term for the libertarian advancement of the neoclassic economic doctrine, which relies on the unlimited free play of market forces and the withdrawal of the state from as many areas of society as possible. Neoliberal thinking places markets at the center of all economic and social life and considers state interventions as mostly harmful because free markets can best regulate themselves; according to the underlying ideology, redistribution—for example, through solidarity-based social protection systems—creates perverse incentives by punishing instead of rewarding the economy's winners. Core messages refer to deregulating capital and labor markets, eliminating price controls, lowering trade barriers, and reducing state influence in the economy, especially through privatization and austerity. Neoliberal practice has liberalized the international movement of capital, limited the power of trade unions, and converted workforces into commodities.

The almost ubiquitous spread of neoliberalism has contributed to shifting the prevailing economic system from one of coordinated market capitalism, in which institutions coordinate important economic decisions and functions such as wage setting, bargaining, and management of social programs, toward a type of capitalism in which the state abstains as far as possible from interfering in the economic issues of individuals and society because free markets are believed to best regulate economics and society. Neoliberal capitalism favors the implementation of a regime that promotes rules created by and for private owners of capital, particularly transnational corporations. It has kept democratic governments from asserting rules of fair competition or countervailing social interests and managed to impose rules of competition and intellectual property tilted to protect incumbents and the profiteers of the neoliberalism virus. Neoliberalism has not only shattered state-owned companies and sold off public assets, it has generally subordinated people's lives to dominance by market logic and thinking.^15^ The concept—and even more so, the practice—of public goods plays, at best, a secondary role in the political and economic debate. In addition, the predominance of neoliberal policies has benefited from and, at the same time, reinforced the belief inherent in capitalism that sustained economic growth is the means to achieve human progress—disregarding the social, societal, and environmental costs and the unrestrained expense of natural, human, and other resources.

Neoliberalism is commonly considered an economic theory. In fact, however, it has become a rather toxic ideology. The neoliberalism pandemic causes millions of premature and preventable deaths year after year through structural adjustment programs in Africa^16,17^ and austerity policies,^18^ as well as its “normal” performance focusing on short-term rent seeking.^19,20^ These are not only the direct and indirect victims of the wars and coups to secure the interests and profits of economic elites. Unemployment, impoverishment, exclusion, and environmental destruction in the service of—and as a consequence of a supposed increase in—economic efficiency, as the pursuit of profit maximization is euphemistically called, promote the spread of chronic as well as infectious diseases and claim far more lives than COVID-19. Understanding these connections is certainly more demanding than reproducing figures and warnings from virologists and other ubiquitous experts with regard to the COVID-19 pandemic,^21^ but no less correct at all.

## Neoliberalism Disease and Global Health

Having said this, it becomes clear that the pandemic caused by the neoliberalism virus has been and is a public health challenge in itself.^22^ Containing this virus is indispensable for improving people's health worldwide and therefore has to be an intrinsic core task of global health. As a serious and ultimate threat to global health, viral neoliberalism is definitely a relevant yet still underestimated topic for health security. Being key drivers of the syndemic, neoliberal logics have contributed to a predominant focus on biomedical challenges and solutions to the COVID-19 pandemic, while relevant environmental conditions and determinants are insufficiently taken into account in both the health sciences and the political debate on how to deal with COVID-19.^20^

The commercialization and privatization of health, medical, and related research, with its impact on the conduct of bioscientific knowledge-making as a key feature of neoliberalism, has long been in the interest of scholarship.^23–25^ The neoliberal science policy prevailing in the global research agenda tends to neutralize political debate by demanding sound evidence instead of a self-correcting marketplace of ideas.^25^ The growing penetration of a neoliberal market logic and entrepreneurial practices into academia is part of a deeper, cultural–ideological process that has increasingly engulfed life sciences.^26^ Considerable literature is available on the implications of neoliberalism for pharmaceutical research, medicine, and health care.^7,27^

It is not only about the overriding power of big pharma and the increasing pervasion of academia by marketization and economization leading to “neoliberal universities”.^28^ Rather, the neoliberal preoccupation with the individual has laid the crucial groundwork for biomedical solutions. Disguising the prescription of biomedical solutions and the wider socioeconomic conditions of neoliberalism is not the result of scientific research in a domination-free world but rather a process arising within particular socio-political contexts and realities of global capitalism that lead to health being increasingly individualized and medicalized.^29^

The biomedical focus tends to overestimate virological and clinical solutions and to marginalize social and human sciences. Given the increasing evidence for the importance of the social, political, and economic determination of health, this reductionism is unjustified vis-à-vis the complexity of global health and can be harmful.^30^ While many scholars and practitioners are aware of the essential need for more complex, multidisciplinary approaches for preventing and overcoming health crises, and while policymakers increasingly include references to the social determination of health in their statements and declarations, this is not yet sufficiently reflected in political decision-making and practice. In the course of the coronavirus pandemic, it became increasingly clear that the almost unchallenged dominance of virologists in the public, media, and policymaking was not backed by adequate empirical evidence and deep comprehension in a situation where theories, assumptions, findings, and observations sometimes emerged as quickly as they disappeared again.

Notwithstanding the immaturity of much of the short-lived evidence, the one-sided focus on biomedical solutions to the COVID-19 pandemic has managed to marginalize other important and meaningful approaches to dealing with such a pandemic. The prevailing biomedical paradigm, and particularly the massive propagation and in some places imposition of immature vaccines with limited efficacy, accompanied by considerable pressure, brought manufacturers with unimagined, state-subsidized billions in profits,^31–33^ substantially favored by the redistribution from public to private and for profit, which is characteristic of neoliberalism: In addition to basic vaccine research being predominantly public, private firms received massive subsidies for applied research, testing, and distribution of the same vaccines and were simultaneously rewarded with weaker regulatory controls.^34^

At the end of the day, the production and distribution of COVID-19 vaccines suggest that the pandemic has reinforced rather than questioned the neoliberal hegemony^35^ and its colonial foundation^36^ and anchored neoliberal thinking even deeper in people's and politicians’ minds. Global vaccine inequity is one of the most outstanding consequences of neoliberal politics.^37^ The fact that low-income countries are forced to compete for the purchase of COVID-19 vaccines and rely to a large extent on the willingness of high-income countries to donate on their behalf^38^ is nothing but another expression of the neoliberal world order imposing competition-driven markets that have long been proven to lead to structural violence and injustice.^39^

Despite all these rather worrying and long-known effects of the neoliberalism pandemic, the articulation of neoliberal logics and the reconfiguring of notions of scientific and social value in both research and (global) health policies remain comparatively unexamined by public and global health scholars. This is surprising given that containing the neoliberalism pandemic would be a crucial step toward improving the public's health worldwide.^21^ Indeed, transferring the dominant approach to fight COVID-19 and the underlying biomedical hegemony in global health to the neoliberal pandemic would consistently raise the claim for an effective vaccine against the neoliberalism virus. Here, unlike coronavirus or other infectious diseases, however, humanity cannot rely on Big Pharma, which definitely belongs to the profiteers of this pandemic. The same applies to philanthro-capitalists such as the Gates Foundations or the Wellcome Trust, which will have no interest in removing the basis of their prosperity. And, unlike COVID-19, the hitherto rather little-known discipline of virology, which has supplanted all other disciplines since the outbreak of the coronavirus pandemic, will be able to contribute very little, at best. Rather, there is a need for a multidisciplinary approach that includes other medical disciplines and, first and foremost, a broad array of humanities and social sciences. The fact that the pandemic had enormous impact on social, economic, environmental, and other domains of human life was not adequately reflected in COVID-19 policies.^40^ As a matter of fact, coercive government responses to the pandemics led to resentment, mistrust, and denial of reasonable measures.^39,41^ Likewise, disease-focused pandemic responses may clash with local priorities, and technical solutions such as targeting the groups most at risk can have undesired effects if context is not understood.^42^

If the effectiveness of immunization measures to contain the coronavirus pandemic has already been rather limited, close to nothing is to be expected from vaccines to protect the world from the neoliberalism virus, and even less from its most recent mutation, the corporate world domination variant. Different from the corporate imperialism virus declared dead,^43^ the new mutation creates a new form of imperialism that overcomes the distinction between high- and low-income countries, between the Global North and the Global South, by enabling corporations to implement a policy of extending power and dominion and to gain economic and subsequent political control of all areas of society in all countries.

## Leading Symptoms of the Neoliberalism Disease

The neoliberalism virus has proven to be extremely efficient in both manipulating individuals and societies and blurring analytics. The virus has a double stranglehold on a large part of the media, in terms of both content and the economic framework conditions.^44^ Fake news, which was so often imputed and deplored in connection with the COVID-19 pandemic, is systematically created and reproduced by influential think tanks, academia, and the media when it comes to neoliberal economic beliefs.^45^ The global context determined by post-truth rules had started long before this term was broadly discussed by the general public during the Trump administration in the United States. Harry Frankfurt described the promotion of myths and half-truths as a kind of intentional nonsense produced without concern with the truth; faking things does not mean that mythmakers themselves necessarily get them wrong.^46^ The impact of neoliberal managerialism, framed by the euphemistic language about healthy competition, freedom, and individual responsibility, tends to be even stronger and more powerful.^47^

Viral neoliberalism has been very successful in concealing the contradiction between economic liberalization and the extension of free markets on the one hand, and the growth in market power and dominance of corporate entities and monopolies on the other.^15^ In fact, under the rules of the game set by neoliberal strategists, pre-existing inequalities have increased and new ones emerged. This is not at all surprising as the “free play of market forces” did not take place in a space free of domination. The recently revealed irregularities that occurred in one testing laboratory for Pfizer's COVID-19 vaccine, and particularly the downplay reaction of government bodies, again prove the dwindling control of the public sector over transnational corporations.^48^ Under the rules of viral neoliberalism, politics, society, and the economy are exposed and subordinated to widely asymmetrical power relations. Lowering trade barriers, deregulating public control and labor relations, and subordinating common goods to profits tends to favor the strongest and most powerful, but also the most ruthless and courageous corporations compared to others, where daring and unscrupulousness are often very close together.

Undoubtedly, the nation state has passed its zenith. The governments of practically all nations have been more or less openly subverted by transnational corporations. Beyond the general openness of politicians toward economic investments of national and increasingly transnational corporations expecting work places and other societal benefits, this can also be observed in the field of health care and health systems. The literature points to five specific negative impacts, namely, rising global inequality, reduced wages and increased debt, redistribution of profits toward the finance sector, growth of personal and financial insecurity, and the relentless commercialization of many areas of social and planetary life, including health systems.^49^

Evidence shows that neoliberal reform approaches in the health sector tend to have negative impacts on key global health issues such as equity and fairness. Among many others, this applies to structural adjustment programs implemented since the 1970s in low-income countries,^50^ the 1981 market-driven health-sector reform in Chile,^51^ various attempts to reform the British National Health System,^52^ and many countries, particularly in the Global South.^53^ The many examples of direct involvement of transnational corporations, especially those in the food industry, show how effective neoliberalism has become and has long been in public health policy.^54^ The increasing commercialization of medicine and the prioritization of private (profit) interests over public objectives and policies, which had already deteriorated people's health and well-being before the COVID-19 outbreak, turned out to negatively affect countries’ capacity to deal with the pandemic^55^ and even tend to hinder effective action against COVID-19.^56^

The somewhat surprising new etatism observed during the initial outbreak of the COVID-19 pandemic turned out to be short-lived. It not only failed to halt the unstoppable march of transnational corporations, rather it refrained from imposing restrictions on them. Political decision-makers have either embraced corporate neoliberalism willingly, or they have been intimidated by the threat of corporate retaliation when trying to govern in the public interest.^57^ A manifesto published almost a decade ago in the *Lancet* shows that concern about the globally unjust economic system being a root cause of current problems is by no means new, and that the insinuated alternative has not yet taken hold: “Our tolerance of neoliberalism and transnational forces dedicated to ends far removed from the needs of the vast majority of people, and especially the most deprived and vulnerable, is only deepening the crisis we face”.^58^

The open, tacit, or coward subordination to the interests and demands of the unrestrained spreaders of the neoliberalism virus has allowed the most influential global players to become the big winners of the pandemic, which has further contributed to increasing extreme richness and broadening inequalities in and between countries.^59^ Returning to the metaphor of the viral neoliberalism, the parallels with respect to CoV-SARS-2 are striking. COVID-19 is negatively impacting a huge share of humanity, particularly the lower socioeconomic strata of the world population, and at the same time benefiting a minority of people, mainly in the Global North, for whom COVID-19 has brought unimagined income opportunities combined with relatively low individual risk.

## Effects on Global Health

Powerful charitable organizations are increasingly shaping global health policies and strategies. Venture philanthropies—the biggest among them the Bill and Melinda Gates Foundation and the Wellcome Trust—have become important actors and drivers in global health. Private-sector billionaires have managed to implement a real philanthro-capitalist regime contributing an ever-growing share of funding for global health. Along with providing enormous financial resources, venture philanthropies use and heavily promote their own approaches. With their billion-dollar donations, the philanthropic foundations drive public policy worldwide. Likewise, they heavily influence academia, science, research, and health care supply worldwide. In applying business concepts and market based solutions to current global health challenges—and due to their sheer financial power—they permeate public policy and people's minds with neoliberal thinking and systematically impose marketization on humanitarian aid and development cooperation. This is not surprising since charitable global health actors are closely entangled with powerful transnational corporations, including Big Pharma, medical device producers, and giants such as Google, Microsoft, and Apple.

The declared aim of the foundations is to help the poor and disadvantaged of this world to a better life. However, their charity often benefits the wealthy and philanthropists themselves. For example, the Wellcome Trust holds shares in the Swiss pharmaceutical companies Novartis and Roche, which produce promising drugs to treat severe COVID-19 infections.^60^ The Bill and Melinda Gates Foundation can claim to have made a decisive contribution to vaccine development and thus to the containment of the COVID-19 pandemic with its $50 million transfer to the Mainz-based manufacturer BioNTech.^61^ On the other hand, this also turned out to be a financial injection into the pharmaceutical company Pfizer, which distributes the vaccine worldwide. The U.S. company is taking unrestrained advantage of the pandemic's plight and is selling the BioNTech vaccine at inflated prices, although public funds in the hundreds of millions have flowed or are still flowing into its development.

The yielding of political decision-makers to the de facto pressure of transnational corporations is also illustrated by another phenomenon. Direct-to-consumer advertising of pharmaceuticals has long been criticized, with the United States and New Zealand being the only high-income countries officially allowing medicine advertisement to the general public.^62,63^ Now, the pandemic has opened up unprecedented opportunities for the vaccine-producing industry to undermine the ban on direct-to-consumer advertising. Until now, this trend has generated little attention among scholars and policymakers. General public concern and insecurity vis-à-vis a new and apparently dangerous health threat and the huge demand for reliable information meets with the need for politicians to implement and justify effective preventive measures. This risky mix has been grinding the wall to protect laymen and laywomen from unproven and often premature information. Journalists eagerly took up all the news and details communicated by vaccine manufacturers and spread them in the general public. The Mainz-based company BioNTech, together with its big transnational ally Pfizer, was particularly effective among the German and other European populations.^61^ COVID-19-related press news issued since early 2020 about the alleged or measured effectiveness of the vaccine Comirnaty were directly taken up by both policymakers and the press. The resulting positive bias toward the BioNTech vaccine among the German and other European populations^64,65^ has certainly contributed to flush substantial profits into the coffers of BioNTech and Pfizer. Particularly worrisome is the fact that this special form of direct-to-consumer advertising puts pressure on policymakers to disrespect scientific recommendations or even make decisions without evidence and thereby further weakens the position of public policies in relation to neoliberal managerialism and entrepreneurship.

The market-oriented neoliberal thinking and practice in international cooperation has materialized in so-called public–private partnerships (PPP), which have become an omnipresent tool in global health. There has been a veritable explosion of such hybrid initiatives between government and non-government entities,^66^ particularly between public entities and philanthropic foundations. Governments and UN organizations are happy to get access to new sources of funding for health, but investments in global health have first and foremost become a business case. Vis-à-vis the sheer financial power of private donors and the dominance of business administration over human and social needs, charitable billionaires have been able to increasingly set the agenda in international cooperation. The public sector in both high-income donor and low-income recipient countries has a rather weak role to play in PPPs. This asymmetry creates serious governance challenges, which are exacerbated by the lack of accountability posed by the structure and procedures of philanthro-capitalists.

PPPs were initially adopted as mechanisms to address the financial gap in health care delivery; however, they have increasingly become vehicles to open up health markets to the private sector. Global health PPP focus on selected disease silos and predominantly on infectious diseases,^67^ and they privilege technological and bio-medical solutions over strengthening health systems and addressing structural determinants of health. Hence, it is not surprising that PPP and philanthropic charity are not capable of effectively contributing to overcoming the global structural inequality and injustice. With the enormous resources invested according to the PPPs’ priorities in global health rather than in real needs, philanthropic foundations and PPPs threaten to undermine the efforts of the global community to improve global health results because they leave, at best, the barriers to achieving health for all untouched, namely, the inequitable distribution of social determinants of health, especially poverty and social exclusion. A sad example from recent history is the world's inability to confront the COVID-19 pandemic by ensuring an equitable distribution of vaccines to poor countries. This is particularly striking because the global community has specifically created an initiative called COVID-19 Vaccines Global Access (COVAX) to ensure global supply. However, COVAX as a kind of super-PPP amplifies governance, accountability, and profit-driven challenges of philanthropy-supported initiatives and demonstrates the extent to which PPPs have become a default solution to fighting global health problems.^68^ Notwithstanding this blatant failure and the fact that super-PPPs meet all the criteria of a super-spreader of the neoliberalism virus, philanthropy is successfully delivering a form of compensatory moral fig-leaf to cover up the enormous inequalities not only in global vaccine access.^20^

## Conclusion and Outlook

The inequitable global distribution of COVID-19 vaccines has highlighted with unusual clarity the extent to which the symptoms of the neoliberal virus are shaping today's world. While overall global wealth has significantly grown, the share of low-income countries that are home to 8 percent of the world's population has remained below 1 percent, and in 26 countries, mostly in Sub-Saharan Africa, per capita wealth has stagnated or even declined.^59^ At the same time, the socioeconomic gap within countries has widened.^69^ In addition, the perversity of an extreme concentration of global wealth is reflected in the fact that the number of billionaires has doubled in the past decade; today, 2,200 billionaires accumulate more wealth than 60 percent of the world population.^70^

No doubt, this blatant injustice is a symptom of the neoliberalism disease, which has long since proven its chronic progressive course. It is still an open question whether human resilience can be sufficiently improved by, for example, training the human immune system or stimulating antigen-specific T cell or other immunologic responses. Transferring the approaches taken to fight the SARS-CoV-2 pandemic, developing a new type of vaccines is definitely required for protecting the world population from ever more detrimental effects of viral neoliberalism. Achieving herd immunity by a mix of measures, including vaccination, is certainly the only way to eliminate the neoliberalism virus. This is of course an extremely demanding undertaking in view of the unequal distribution of financial resources and power, a kind of updated form of David vs. Goliath.

Sustainability might be a promising approach for pushing back viral neoliberalism. The fear of irreversible damage to the earth and deterioration in the quality of life worldwide have become global concerns. There is growing awareness of the existing productive systems and consumption patterns threatening the continuity of the existing living conditions. The inequitable and undemocratic nature of the current economic system is unsustainable because it endangers the planet's possibilities for supporting and reproducing life. To enable sustainable living, it is ultimately inevitable to impose political limits on corporate power, because even broader and stronger mass protest movements will be unavailing as long as governments refrain from tackling and shackling the corporate miscreants.

## List of abbreviations

COVAX: COVID-19 Vaccines Global Access
COVID-19: Coronavirus Disease 2019
HIV/AIDS: Human Immunodeficiency Virus/Acquired Immunodeficiency Syndrome
IMF: International Monetary Fund
PPP: Public-private partnership
SARS-CoV-2: Severe Acute Respiratory Syndrome Coronavirus-2
UN: United Nations
UNICEF: United Nations International Children's Emergency Fund
WHO: World Health Organization
WTO: World Trade Organization
